# Multiple Chemical Sensitivity and the SLC Gene Superfamily: A Case–Control Study

**DOI:** 10.3390/ijms26136484

**Published:** 2025-07-05

**Authors:** Esther Alcorta, Carolina Gomez-Diaz

**Affiliations:** NEUROSEN Group, Faculty of Medicine, Department of Functional Biology, Institute of Neurosciences of the Principality of Asturias (INEUROPA), University of Oviedo, c/Julian Claveria, 33006 Oviedo, Spain; ealcorta@uniovi.es

**Keywords:** multiple chemical sensitivity, chemical intolerance, whole-exome sequencing, *SLC* gene superfamily, sensory hyperexcitability, olfactory hyperactivation

## Abstract

Multiple chemical sensitivity (MCS) is a disease of unknown etiology with multiple symptoms. Triggered by exposure to environmental chemicals, it results in multiorgan effects. Studies on MCS use different approaches, ranging from searches for environmental triggers to susceptibility genes. Genetic research deals with genes for chemical detoxification, oxidative stress, inflammation, and neurodegeneration, as well as immune function and mast cell activation, with uneven results. The sensory hyperexcitability symptom has not been studied yet but has recently been linked to a member of the *SLC* gene superfamily. To explore its role in MCS disease, a complete-exome analysis was performed in a small number of subjects. Low-frequency genetic variants were analyzed for each individual, and their homozygous or heterozygous presence was determined in four groups of genes related either to the *SLC* superfamily members or to previous studies in MCS. We found homozygous rare variants in affected individuals only for the *SLC* gene superfamily, where each patient had at least one. Variants in heterozygosis and certain SNPs also point to *SLC* genes related to neurotransmitter synthesis, release, and clearance, as well as to the level of cellular excitability, as potentially underlying the differences.

## 1. Introduction

Multiple chemical sensitivity (MCS) is a disease of unknown etiology with multiple symptoms. It is characterized by an overreaction to chemical compounds at concentrations that are harmless to other people [1]. It presents a whole battery of symptoms that have been associated with various organs and functions, such as sensory, respiratory, gastrointestinal, dermatological, neurocognitive, and even psychological effects, among others. Exaggerated reactions and the associated effects disappear when the chemical substance that causes them disappears [1,2,3,4]. MCS usually manifests in adults and is more frequent in women than in men [4]. During the last 40 years, a series of studies ranging from the search for environmental triggers to susceptibility genes have tried to delimit its characteristics, the possible origin of the adverse effect, and its epidemiology and treatment [5,6,7,8].

Genetic research has attempted to associate genetic variants in patients with MCS to some of the described processes implicated in MCS, such as chemical detoxification, oxidative stress, inflammation, and neurological disorders, with unequal results [2,9,10,11,12,13,14,15,16,17]. On the other hand, a blind search for significant genetic variants in a genome-wide study found positive correlations between single-nucleotide polymorphisms (SNPs) and MCS-affected individuals. In the study, some risk and protective SNPs pointed to immune function and mast cell activation as part of the biological processes involved [18]. However, there is still a long way to go before the complete biological pathway underlying MCS is unraveled.

A recent study including a principal component analysis of symptoms revealed that the most frequent symptom was hyperosmia (96.9% of patients), followed by asthenia (82.9%) and dyspnea (81.5%) [19]. Olfactory hypersensitivity has been correlated with some biological differences in the detection and processing of this information by the brain in patients with MCS [20,21,22,23,24,25,26] and has even been pointed out as the primary criterion for diagnosis by some authors [21]. Nevertheless, to date, no genetic research has specifically studied the relationship between hyperosmia and MCS.

A study in the model species *Drosophila melanogaster* reported hyperosmia by silencing, in glial cells surrounding the soma of olfactory receptor neurons, the *eaat2* gene, which encodes an excitatory amino acid transporter. Moreover, modifying the excitability level of these glial cells by selective ion input also modifies olfactory perception, both increasing and decreasing it [27]. Considering that the *Drosophila eaat2* gene has a main orthologue in humans, the *SLC1A2* (solute carrier 1A2) gene, a glutamate transporter, and a moderate homology with other genes of the same family (OrthoDB v12.0, NCBI ID 33247), we have paid attention to this gene superfamily in our human sample. Considering the possible genetic heterogeneity behind MCS, we extended the study not only to the genetic orthologues of the *eaat2* gene but also to other functional homologues.

The *SLC* superfamily of human genes includes 66 gene families (more than 400 genes) encoding membrane transporter proteins that allow for the passage of ions and small molecules, such as neurotransmitters and amino acids, mainly across the cell membrane [28,29]. As such, it affects numerous basic functions of the organism. Among the different solute transporters, some are responsible for the removal of neurotransmitters from the synaptic cleft, while others are related to the synthesis and release of these molecules into the synapse, as described in the REACTOME knowledge base (https://reactome.org (accessed on 2 April 2025)) [30,31,32]. However, due to the symporter and antiporter properties of some SLC proteins that transport solutes along with other ions, SLC proteins that affect cellular excitability through only ion transport [33] may also have an effect, and they are also studied here.

In this article, to explore the relationship between MCS and the *SLC* gene superfamily, we performed whole-exome sequencing (WES) in a small group of six MCS patients and five control individuals. Low-frequency genetic variants (<5% of the population) were analyzed for each individual, and their homozygous or heterozygous presence was determined for four groups of genes related to members of the *SLC* superfamily or to previous studies in MCS. Group A refers to 17 genes encoding detoxification and oxidative stress proteins [9,10,11,13,14]. Group B includes 29 genes encoding members of the transient receptor potential (TRP) gene family of ion channels, which have been linked to neuronal degeneration in MCS [3,17]. Group C includes members of the SLC gene superfamily, and Group D previously reported 19 genes with risk and protective SNPs associated with MCS derived from a blind GWAS study [18]. The idea behind the analysis of the four gene groups is to determine whether any possible difference patterns we could find between patients and controls for the SLC gene superfamily are common or rare in relation to the other three groups, considering the large number of genes analyzed and the small sample size.

In addition to the analysis of gene variants (rs) and their homozygous or heterozygous character in each individual and for each gene group, we checked the frequencies of variants grouped by gene families and the presence of common variants among patients and controls to compare both groups. Also, we checked whether the changes associated with each variant corresponded to a regulatory region of the gene or to the coding sequence of the protein, either as a synonymous or non-synonymous protein change. Finally, SNP (rs) analysis among patients sharing rare genetic variants that do not appear in the control group was performed.

## 2. Results

The lack of knowledge on the mode of action (additive or dominant–recessive inheritance) of the gene alleles putatively involved in susceptibility to MCS prevents us from having a clear idea a priori of the results that may represent a positive indication. Nor can we initially rule out results by type of gene variant, because a change in the encoded protein can have an effect, but a change in a regulatory region or even a synonymous change in the coding sequence can also have consequences on the regulation of gene expression.

In this scenario, we distinguished the differences in homozygosis and heterozygosis and, as far as possible, we compared the groups of patients and controls to obtain an overall picture.

### 2.1. Whole-Exome Analysis

A global study of the genetic variants present in the exome (around 20,000 genes) of six patients and five control individuals was performed for three different situations: (a) one in which we only applied quality control filters to the sample, (b) one in which we added a filter for low-frequency variants (<1% of the population) that involve changes in the coding sequence to the quality control, and (c) one in which quality control was followed by a filter for low-frequency variants (<1% of the population) that involve changes in non-intronic regions.

In none of the three situations (Table 1) were there statistically significant differences between the patient group and the control group in terms of the number of genetic variants observed in the whole exome.

Therefore, if there is a genetic origin in the appearance of MCS, it cannot be due to a generalized increase in rare variants in the genome of the patients compared to controls.

### 2.2. Low-Frequency Variants (<5% of the Population) in the A, B, C, and D Gene Groups

#### 2.2.1. Homozygous Variants in Participants

We studied rare genetic variants (<5% of the population) in homozygosis in the four gene groups (A–D), as listed in Section 4, indicating the gene in the observed cases. The idea is that whether the MCS susceptibility allele acts in an additive or dominant–recessive manner, it will manifest itself in homozygosis. The incidence in patients was compared with that in controls (Table 2).

Individuals with homozygous variants are very scarce or nonexistent in our small sample of genes from Groups A (17 genes), B (29 genes), and D (19 genes). Only one appears in Group B, among the controls, and it corresponds to a synonymous change in the base of an exon (CS). In Group D, rare homozygous variants appear for neither the putative 11 risk SNPs studied nor the putative 8 protective SNPs.

However, in Group C of the solute carrier superfamily, in which more homozygotes appear, as would be expected from the study of more than 400 genes, there is a clear imbalance between the group of controls and patients, with a statistically significant higher number of rare variants (*p* < 0.01) among those affected. In fact, all affected individuals show at least one, and in some cases two, gene variants of the *SLC* gene superfamily in homozygosis. Although no two patients show homozygosity for variants of the same gene, homozygosis for members of the same gene family—*SLC6A1* and *SLC6A14*, as well as *SLC13A3* and *SLC13A4*—occurs in several patients.

The variants are located almost equally in regulatory and coding regions of the gene, producing, in the latter case, either synonymous or non-synonymous changes in the resulting protein.

The *SLC6* gene family is a sodium- and chloride-dependent neurotransmitter and amino acid transporter. SLC6A1 transports GABA, and SLC6A14 transports neutral and cationic amino acids, such as glycine. Both GABA and glycine are very important inhibitory neurotransmitters in the brain and spinal cord, although they are not limited to them [34,35,36]. Both SLC6A1 and SLC6A14 are expressed not only in neurons but also in glial cells and may therefore act as neuromodulators. Moreover, some inhibitory synapses can corelease GABA and glycine [37].

Meanwhile, *SLC13* corresponds to the gene family of sodium sulfate/carboxylate cotransporters. Both the sodium-dependent dicarboxylate transporter (SLC13A3) and the sulfate transporter (SLC13A4) show a wide tissue distribution, with most of them being expressed in the epithelial cells of the kidney and the gastrointestinal tract [38].

The other homozygous variants in patients are in *SLC4A2*, *SLC9A7*, *SLC35D2*, and *SLCO1A2*. *SLC4A2* encodes a membrane transport protein that exchanges Cl^−^ and HCO_3_^−^, contributing to cellular pH homeostasis [39]. The *SLC9* gene family is a sodium–proton antiporter that transports Na^+^ into the cell and H^+^ out of the cell [40]. *SLC35* is a nucleotide–sugar transporter gene family [41,42]. One homozygous variant of *SLC35A2* appeared in a control individual, and one of *SLC35D2* in a patient. Finally, *SLCO1A2* (from the *SLC21* gene family, subfamily 1) is a solute carrier organic anion transporter mainly expressed in the liver [43].

#### 2.2.2. Heterozygous Variants in Participants

As previously indicated, variants in heterozygosis for MCS susceptibility genes will only manifest their effect if we are dealing with a dominant or additive expression pattern (in this case, to a lesser extent than for the homozygote), but not if it is a recessive variant.

##### Group A

Rare heterozygous gene variants (<5%) of 17 genes related to detoxification and oxidative stress were analyzed in the sample of six patients and five controls. The frequency of heterozygous variants is shown in Table 3.

The small number of samples does not allow us to obtain reliable statistical data on a gene-by-gene basis. Only in one case did we observe an appreciable difference in the number of variants between patients (1) and controls (6) for rare variants of the *CYP2D6* gene. However, more rare variants appear in the control group than in the patient group, contrary to what we would expect if the rare variants corresponded to susceptibility genes.

Overall, we also did not observe a greater tendency for rare variants to present in the patient group compared to the control group for group A genes in heterozygosis.

##### Group B

In this group, we studied rare variants in heterozygosis for 29 genes encoding TRP-type ion channels (Table 4).

No significant differences were found for this group of genes between controls and patients. However, the higher frequency of heterozygous variants in the patient group (12) for the *PKD1* gene versus the number of variants in the controls (6) is noteworthy, although we cannot determine its statistical significance. The *PKD1* gene encodes an integral membrane protein that functions as a regulator of calcium-permeable cation channels and intracellular calcium homoeostasis [44].

##### Group D

Here, we studied the low-frequency variants (<0.05% of the population) in heterozygosis for the 11 genes with putative risk variants and for the 8 genes with putative protective variants found in a previous GWAS report on MCS [18]. The results are presented in Table 5.

There are no appreciable imbalances between patients and controls for rare variants in these genes, but there are insufficient data for a gene-by-gene analysis. There are also no significant differences between the patient and control groups when the data are taken together.

##### Group C

The analysis of rare variants (<5% population) in heterozygosis in the *SLC* gene superfamily, including the typical *SLC* genes and other atypical genes that preferentially use other names different to *SLC*, returned 291 variants in our sample of five controls and six patients. Table 6 shows the heterozygote frequency data as a whole to examine their statistical significance.

Although, when we analyzed the frequency of homozygotes in rare variants for Group C genes, there was clearly a statistically significant imbalance between patients and controls (*p* < 0.01), this was not the case when we analyzed heterozygotes. Firstly, many more rare variants appear in heterozygosis in both controls and patients and affect many more genes of the *SLC* superfamily, so it is possible that the differences in the genes that are most important for their function in the occurrence of MCS and that may have an effect even in heterozygosis are diluted among the rest.

According to the REACTOME database (https://reactome.org) [30,31,32], the list of *SLC* genes of interest was restricted to those that most directly influence neurotransmitter synthesis, release, and clearance, which was our starting hypothesis due to functional homology with the *eaat2* gene of *Drosophila melanogaster*. Table 7 shows the low-frequency variant data for the restricted list of *SLC* genes in heterozygosis grouped by gene family.

We found highly statistically significant differences (*p* < 0.01 **) in the frequency distribution of rare variants in heterozygosity between patients and controls for the *SLC* gene families most directly involved in the processes of neurotransmitter synthesis, release, and clearance in the synaptic cleft.

The type of changes (R, SC, NSC) in rare variants in heterozygosis in the restricted List C was also analyzed in both the patient and control groups (Table 8).

For the group of genes on the restricted List C, related to neurotransmitter release and clearance, in the control group, the heterozygous variants appear preferentially in regulatory regions of the gene, but this is not the case in the patient group. Differences between patients and controls become statistically significant (*p* < 0.01 **) for synonymous variants in the gene coding region and are close to significance for the non-synonymous variants in the coding region that result in an amino acid change. This result may indicate that the effect of the change in the coding site is not only at the gene product level but also at the DNA sequence level, and this may have an effect on the regulation of gene expression.

### 2.3. Common SLC Gene Superfamily Variants Among Patients

**Figure 1:**
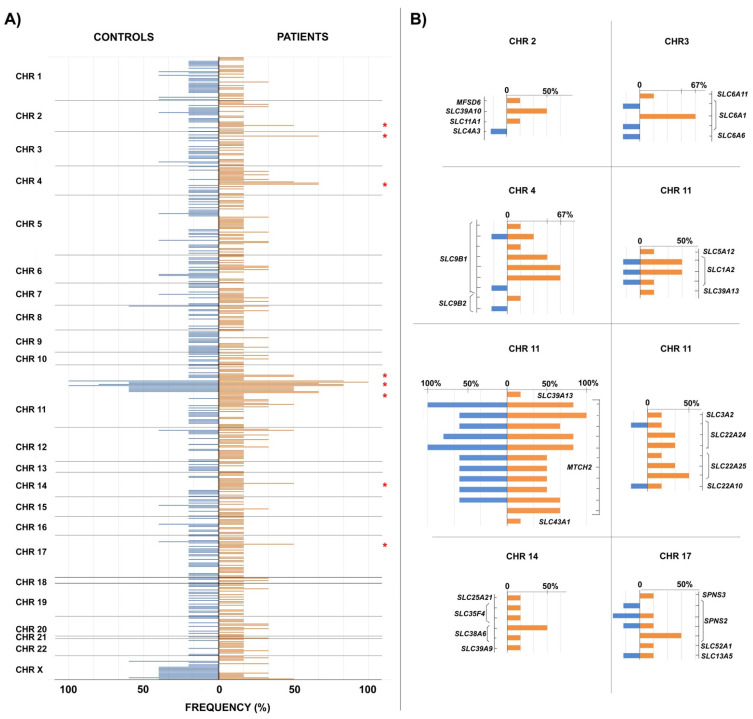
SLC gene superfamily variant distribution among patients and controls. In panel (**A**) Figure 1 shows the frequencies (in percentages) of the total number of *SLC* gene superfamily variants observed in the control group (in blue) towards the left of the vertical axis, while those of the patients are drawn to the right of the axis (in orange). When the same variant appears in both control individuals and patients, the bases of the corresponding bars coincide on the position of the vertical axis. Panel (**B**) shows in detail the regions marked with a red asterisk in panel (**A**). The positions of the variants on the vertical axis are ordered relative to each other but are not scaled.

In this section, we analyze the existence of common genetic variants, either in homozygosis or heterozygosis, between the controls and patients in our sample. Since we are not dealing with a single gene but with variants (SNPs) of genes from different families within the large superfamily of *SLCs*, we do not expect to find absolute answers, but we look for indications of relatively frequent variants in our sample of patients which do not appear in the control sample (Figure 1).

As can be seen, the genes of the *SLC* superfamily occupy positions on all chromosomes of the human genome. Although most of the rare variants described appear in only one individual, both in the control group and in the patient group, in some cases, they are shared by several individuals in the sample. We analyzed in detail those cases in which variants shared by at least three patients (50% of the patient sample) were observed, looking especially for cases in which the same variants did not appear in any control individual. These would be the cases in which there is a higher probability that the variant is associated with the appearance of MCS.

The region that is most striking at first glance is located on chromosome 11 and reaches maximum frequency values in our sample. However, it is not an informative region for our analysis, since it gathers several variants shared in both controls and patients. The reason we found all these variants in our sample, despite the filter for rare variants (<5%), is because for four variants, there is no population frequency data available in the database, so they had not been filtered against, and in other cases, the frequencies are not so rare, close to 5%. They belong to the *MTCH2* gene, also called *SLC25A50*, and correspond to a mitochondrial transporter [45].

On the other hand, in the right half of panel (B) and included in chromosome 11 is the detail of two variants of the *SLC1A2* gene that, although they appear in 50% of the patients (three individuals), also appear in one individual in the control group. Although these variants did not initially meet the conditions that we established for our detailed analysis, we focused on them because this gene is the genetic orthologue with the greatest similarity to the *eaat2* gene of *Drosophila*, whose malfunction in some glial cells produces olfactory hyper-reactivity [27].

The other six sections in panel (B) show variants that appear in at least three patients and no control individuals. In some cases, this occurs for a single variant of the gene, and in others it corresponds to several variants of the gene in nearby regions, which could lead to the same functional consequences.

The list of variants with these properties, including their frequency in the general population and the gene to which they correspond, is given in Table 9.

Three genes in this group—*SLC6A1*, *SLC22A25*, and *SLC38A6*—belong to families of the restricted List C, related to the synthesis, release, and clearance of neurotransmitters. SLC6A1 transports GABA through the cell membrane [46,47,48]. *SLC22A25* encodes a plasma integral membrane protein which functions both as an organic cation transporter and as a sodium-dependent high affinity carnitine transporter, and it is involved in the active cellular uptake of carnitine [49]. *SLC38A6* enables L-glutamate transmembrane transporter activity and L-glutamine transmembrane transporter activity [50], with an important role in cerebellar glial and neuronal metabolism [51].

### 2.4. Gene Ontology Enrichment Analysis of Candidate Genes

Although, in this report, we focus on the SLC (solute carrier) gene superfamily as potential candidate genes for MCS, and they already have known associated functions, an additional Gene Ontology enrichment analysis was performed to further refine the functional interpretation of the selected genes. The analysis was based on a list of 13 *SLC* genes derived from Table 2 and Table 9 (please see Section 4).

**Figure 2:**
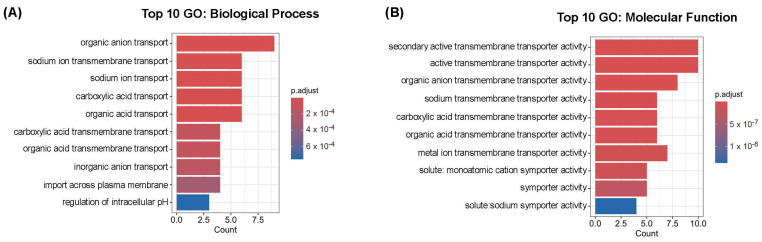
Gene Ontology enrichment analysis of SLC candidate genes. Top 10 enriched GO terms in the Biological Process (BP) category (**A**) and in the Molecular Function (MF) category (**B**). The input consisted of 13 human solute carrier (SLC) genes from Table 2 and Table 9. Bar plots display the most significant GO terms ranked by adjusted *p*-value.

The GO analysis for Biological Process (BP) revealed significant enrichment in categories related to the transmembrane transport of different types of ions, as expected based on the known roles of *SLC* genes, along with processes such as the regulation of intracellular pH (Figure 2A). For Molecular Function (MF), the most enriched categories were also consistent with the nature of these proteins, highlighting terms such as transmembrane transporter activity and symporter activity (Figure 2B), which are characteristic of SLC-mediated transport.

## 3. Discussion

Multiple chemical sensitivity (MCS) syndrome has been defined in terms of the symptoms it presents, although its etiology is unknown. These symptoms affect multiple organs and encompass a range of different phenomena, including hypersensitivity to certain chemical compounds, inflammation, oxidative stress, neurological disorders, and even immune function. Attempts to find a common genetic cause have allowed some genes to be associated with the condition in particular studies, but these have not been confirmed in new studies [12]. All this suggests that we are probably dealing with a complex syndrome which brings together a group of conditions of varied genetic origin and that its presence is not due to a single gene but to a combination of several genes with major effects. If we add to all this the fact that we can obtain different effects with modifications in different gene regions and coding and regulatory areas, and that we can have a different gene–dose effect depending on the particular allele of the gene, according to its dominant, recessive, or additive character, we obtain ranges of variability that are difficult to manage.

With this idea in mind, we approached the detailed genetic study of the whole exome in a sample of six patients and five controls, looking for rare variants that could explain the low frequency of MCS in the population. As in other studies (see, for example, [13] and the genes for the metabolism of xenobiotics/endobiotics), we focused on genes related to one of the symptoms of MCS, a major symptom (present in 96.9% of the patients [19]) that had not previously been explored from a genetic point of view: olfactory hypersensitivity. This hypersensitivity does not imply a lowering of the odor detection threshold (see review [52]); instead, low or normal odorant concentrations are detected as high or very high by MCS patients. However, brain responses to threshold odor concentrations are greater in MCS patients than in controls [53]. These data suggest a scenario of hyper-reactivity rather than hypersensitivity [1], with multiplicative effects from reception to perception, which can become harmful to patients.

Based on a similar case of olfactory hyper-reactivity in the model species *Drosophila melanogaster* [27] mediated by the *eaat2* gene, and considering the possible genetic heterogeneity behind MCS, we extended our study not only to the genetic but also to the functional homologues of the *eaat2* gene in humans, the *SLC* gene superfamily. An analysis of three other groups of genes related to MCS in previous reports [2,9,10,11,13,17,18] was also performed as an additional control for comparison because of the small size of the sample (six patients and five controls).

To avoid overlooking recessive genetic modifications important for MCS, the first analysis was performed with the rare variants in homozygosity. We saw a distinct pattern for the four groups of genes studied. Only rare variants in homozygosis were found in the patient group for the *SLC* genes. A single case of the *TRP* ion-channel gene group (Group B) and of the *SLC* genes also appeared in the control group, but the imbalance between patients and controls in this last group was statistically significant (*p* = 0.0036 **) even for such a small sample of individuals. It is important to note that in all patients, there was at least one *SLC* gene with a rare variant in homozygosis, and in two of the six patients, there are two homozygous variants. These data would be compatible with the relationship between the *SLC* gene superfamily and the presence of MCS.

The analysis of rare variants in heterozygosis should reflect only dominant and additive effects of genes, if they exist. Overall, a smaller effect than that observed for variants in homozygosis would be expected if the allele were additive, and no differences would be seen if the rare variant were recessive. Looking at the three groups of genes previously related to MCS, groups A, B, and D, there were no significant differences in the number of rare variants present in the patient group compared to the controls. The probability value was close to significance (*p* = 0.077) for Group A (detoxification and oxidative stress genes), but in the sense of observing more variants in the control group. Overall, for genes in the *SLC* group, no significant differences for variants in heterozygosity between patients and controls were observed either. However, if we focus our attention on those *SLC* genes related to neurotransmitter synthesis, release, and clearance, we observe a significantly higher number of variants (*p* = 0.005 **) in the patient group compared to the control group. Again, the results are compatible with an effect of *SLC* genes in MCS syndrome, especially in the group related to synaptic transmission through neurotransmitters, which are probably more susceptible, even in heterozygosis, to functional consequences for the individual.

It could be argued that given the different numbers of genes studied in the four groups (between 17 and 29 for A, B, and D and about 400 for Group C (2% of the genome)), it is logical that more rare variants should be found for Group C than for the other three in the sample. However, the clearly unbalanced distribution of rare variants in homozygosis, with more of them in patients than in controls, cannot be explained by number issues. Furthermore, focusing on heterozygotes and restricting the analysis to *SLC* genes related to neurotransmitter synthesis, release, and clearance, we are only analyzing 12 out of the 66 gene families initially studied. This corresponds to approximately 84 genes, given that the average *SLC* family comprises seven members [54], and we reach the same conclusion.

We made further efforts to find any rare genetic variants (less than 5% of the population) that could be good candidates for causing MCS. To do this, we searched the sample for those SNP variants common to at least 50% of the patients and which do not appear in any control individual. Considering that there are many genetic variants for the same gene, finding rare shared variants in such a small sample of patients is at least suggestive.

Rare SNP variants that meet this condition were found in six *SLC* genes. Among them, we will highlight the case of the rs41293373 variant of the *SLC6A1* gene, with a very low estimated probability in the population (*p* = 0.006309) but appearing in heterozygosis in two of the six patients in the sample and in homozygosis in one more patient but in none of the controls. The variant affects a regulatory region of the gene, the 3′UTR region.

The *SLC6A1* gene encodes a Na^+^- and Cl^−^-dependent transporter of the GABA neurotransmitter and it has been extensively studied (see reviews [35,47]). GABA is the primary inhibitory neurotransmitter in the cerebral cortex and has the highest level of expression in this region [55], while its precursor, glutamate, acts as an excitatory neurotransmitter. Maintaining a balance between inhibitory neural transmission via GABA and excitatory neural transmission via glutamate is essential for cell membrane stability and proper neurological function. Thus, glutamate buffering has been related to oxidative stress and neuropil degeneration in the *Drosophila* brain [56], and glial GABA transporters modulate signaling in epilepsy and stroke [57,58,59]. Moreover, the *SLC38* family, which includes a rare SNP variant in the *SLC38A6* gene listed in Table 9 (SNPs that are common to 50% of the patient group but appear in none of the control individuals), has been related to the glial and neuronal metabolism of glutamate and glutamine in the cerebellum [51].

Several mutations in coding regions of the *SLC6A1* gene have been linked to myoclonic–atonic epilepsy [60]. It should be noted that mutations in the *SLC1A2* gene, an ortholog of the *Drosophila eaat2* gene that encodes a high-affinity glial glutamate transporter, can also cause some types of epilepsy in humans [61].

Particularly intriguing is a case report on MCS in a 23-year-old female patient with a variety of multi-organ symptoms typical of this syndrome but who had also suffered partial seizures since the age of 17, leading to a diagnosis of parietal lobe epilepsy. Administration of levetiracetam, which reduces the release of presynaptic neurotransmitters, including glutamate, eliminated her MCS symptoms [62].

We can ask whether alterations in coding or regulatory regions of the *SLC6A1* gene, such as those found in our sample, or in others from the same family could induce multi-organ MCS symptoms with or without causing seizures. *SLC25A38*, belonging to the *SLC* superfamily, is described as containing a risk variant, according to a GWAS study [18]. It encodes a glycine carrier through the mitochondrial membrane and not through the cell membrane. *SLC1* and *SLC7* are also related to inflammation and autoimmunity [63].

Concerning the affected region of the gene (the regulatory or the coding sequence) in each case, it has been shown that weak DNA methylation around the *SLC6A1* gene promoter is related to temporal lobe epilepsy [64], suggesting that epigenetic changes may be involved in the onset and progression of the symptoms and highlighting the importance of modifications both in non-coding regions and coding regions, even if this involves synonymous changes in the encoded protein, as we have observed in heterozygotes for rare variants of *SLC* genes involved in neurotransmitter synthesis, release, and clearance (Table 8).

A third gene that stands out in our sample is *SLC9B1*, with three rare SNP variants located close together in the genome: rs201645894 (*p* = 0.036757), rs369692318 (*p* = 0.029791), and rs764250836 (*p* = 0.009533). They are present in three or four patients but absent in the controls. All three correspond to protein-coding regions, with the first two located within exons and the last affecting splicing. The gene encodes a Na^+^/H^+^ antiporter that is essential for the pH homeostasis of the cytoplasm and/or organelles [65]. Although its modifications have not been directly linked to any disease, it is easy to imagine a possible potentiating effect on pathologies due to other Na^+^ concentration-dependent transporters, for instance. This could be especially relevant considering that in the patient sample, five out of six patients have one or more of these three rare variants in the *SLC9B1* gene, together with a rare variant in the *SLC6* family of Na^+^- and Cl^−^-dependent neurotransmitter transporters.

Although the evidence presented should be further validated in a larger sample of individuals where more complex statistics can be performed to either support or refute our hypothesis, we believe that the results presented in this report allow us to propose the *SLC* gene family as significant potential candidates for the development of MCS syndrome.

## 4. Materials and Methods

### 4.1. Ethical Considerations

This study complies with the principles established in the Declaration of Helsinki (amend. 2013). All participants recruited for this study provided written informed consent for the collection of peripheral blood samples in EDTA tubes for the research purposes established in the project approved by the Research Ethics Committee of the Principality of Asturias (code CEimPA 2024.289).

### 4.2. Study Cohorts

Eleven individuals were recruited as part of this pilot study. The participants were all adults and agreed to the consent form approved by Research Ethics Committee of the Principality of Asturias. Among the eleven participants, six were diagnosed with MCS, and five were controls, none of whom were genetically related to the patients. Of the six participants diagnosed with MCS, five were women and one was a man. In the control group, two participants were women and three were men. The gender imbalance reflects the higher prevalence of MCS among women than among men, but in such a small sample, we do not expect this to interfere with the results. The participants diagnosed with MCS were between 52 and 70 years old, whereas the age of the control group ranged from 48 to 68 years.

### 4.3. Groups of Genes and Gene Families Tested

Group A includes the following 17 genes previously studied for a possible association with MCS [9,10,11,13,14]: *CAT*, *CCKBR*, *CYP2D6*, *FADS1*, *GSTM1*, *GSTP1*, *GSTT1*, *MTHFR*, *NAT2*, *NOS2*, *NOS3*, *OPRL1*, *PON1*, *PPARG*, *SCN9A*, *SIRT1*, *SOD2*.

Group B includes 29 genes encoding members of the transient receptor potential (*TRP*) gene family of ion channels that have been linked to neuronal degeneration in MCS [3,17]: *TRPA1*, *TRPC1*, *TRPC2*, *TRPC3*, *TRPC4*, *TRPC5*, *TRPC6*, *TRPC7*, *TRPM1*, *TRPM2*, *TRPM3*, *TRPM4*, *TRPM5*, *TRPM6*, *TRPM7*, *TRPM8*, *MCOLN1*, *MCOLN2*, *MCOLN3*, *PKD1*, *PKD2*, *PKD2L1*, *PKD2L2*, *TRPV1*, *TRPV2*, *TRPV3*, *TRPV4*, *TRPV5*, *TRPV6*.

Group C corresponds to the *SLC* gene superfamily, which includes typical *SLC* genes and atypical *SLC* genes, which are given names other than *SLC* but perform the same type of function. This group includes the *SLC1* to *SLC66* gene families, but in some cases, the following names are commonly used: *SLCO* gene family (corresponding to the *SLC21* gene family [43]; *UCP* and *MTCH* (members of the *SLC25* family of mitochondrial carriers); *RHAG*, *RHBG*, and *RHCG* (from the *SLC42* family of ammonia transporters [66]); *FLVCR* (member of the *SLC49* heme transporter family); *XPR1* (member of the *SLC53* family of phosphate carriers); *MPC* (member of the *SLC54* mitochondrial pyruvate carriers); *LETM* family (corresponding to the *SLC55* mitochondrial cation/proton exchangers); *SFXN* family (corresponding to the *SLC56* mitochondrial serine transporter activity [67]); *NIPA* family (*SLC57*, NiPA-like magnesium transporter family); *MAGT* family (belonging to the *SLC58* MagT-like magnesium transporter family); *MFSD2* to *MFSD5* (belonging to *SLC59* to *SLC61*, respectively); *ANKH* (from the *SLC62* Pyrophosphate transporter family); *SPNS* family (corresponding to the *SLC63* family of Sphingosine-phosphate transporters); *TMEM* (*SLC64* family of Golgi Ca2+/H+ exchangers); *NPC* family (belonging to the *SLC65* family of NPC-type cholesterol transporters); *CTNS* and *MPDU1* (both belonging to the *SLC66* family of cationic amino acid exporters).

The Group C restricted list includes genes that encode carriers that have been directly related to neurotransmitter synthesis, release, and clearance in the synaptic cleft, as described in the REACTOME knowledge database (https://reactome.org) [30,31,32]. It contains 12 of the 66 *SLC* gene families: *SLC1*, *SLC4*, *SLC5*, *SLC6*, *SLC7*, *SLC8*, *SLC10*, *SLC16*, *SLC17*, *SLC18*, *SLC22*, *SLC38*.

Group D includes genes with 11 risk and 8 protective SNPs derived from a blind GWAS study [18]: *ANKRD6*, *NALF1 (FAM155A)*, *WWOX*, *DAB1*, *RIMS2*, *DOCK2*, *SLC25A38*, *GIGYF2*, *BCAS3*, *STK32B*, *ECHDC2* and *GRIN2A*, *GNA12*, *CXCR2P1*, *LRIG2*, *TNIK*, *PAK5 (PAK7)*, *TSPAN9*, *ARHGAP22* for the risk and protective groups, respectively.

### 4.4. Isolation of Genomic DNA

Genomic DNA was isolated from 5 mL of peripheral blood samples collected in EDTA tubes using the QIAamp DNA Blood Mini kit (Quiagen, Hilden, Germany) following the manufacturer’s instructions. DNA quality, concentration, and integrity were assessed with QuantiT PicoGreen (Invitrogen, Waltham, MA, USA) and Tapestation gDNA screentape (Agilent, Santa Clara, CA, USA).

### 4.5. Library Preparation and Exome Sequencing

Standard exome capture libraries were created by Macrogen (Seoul, Republic of Korea) with the Agilent SureSelect Target Enrichment protocol for Illumina paired-end sequencing library (Version C2, December 2018) using the SureSelect Human All Exon V8 probe set (Agilent Technologies) following the manufacturer’s protocol. In summary, 1 μg genomic DNA diluted in EB Buffer was sheared to a target peak size of 150–200 bp using the Covaris LE220 focused-ultrasonicator (Covaris, Woburn, MA, USA) according to the manufacturer’s recommendations. Briefly, the microTUBE Strip was loaded into the tube holder of the ultrasonicator and the DNA was sheared using the following settings: mode, frequency sweeping; duty cycle, 10%; intensity, 5; cycles per burst, 200; duration, 60 s × 6 cycles; temperature, 4–7 °C. Then, adapters were ligated to the fragmented DNA and once ligation was assessed, the adapter-ligated product was PCR-amplified following the manufacturer’s recommendations. For exome capture, 250 ng of the DNA library was mixed with the hybridization buffer, blocking mix, RNase block, and 5 µL of the SureSelect All Exons capture library according to the standard Agilent SureSelect Target Enrichment protocol. Hybridization to the capture baits was conducted at 65 °C using a heated thermal cycler lid option at 105 °C for 24 h on a PCR machine. The captured DNA was then washed and amplified. The final purified product was then quantified using qPCR according to the qPCR Quantification Protocol Guide (KAPA Library Quantification kits for Illumina Sequencing platforms), and quality was assessed using the TapeStation DNA screentape D1000 (Agilent).

Whole-exome sequencing of the samples was performed on the Illumina NovaSeqX sequencer platform (Illumina, San Diego, CA, USA) by Macrogen (Seoul, Republic of Korea), with an average of approximately 90 million 150 bp total reads per sample.

### 4.6. Bioinformatic Analyses

Bioinformatic analysis of the raw data was performed by DreamGenics (Gijon, Spain) following standard procedures. Briefly, the processing of the FASTQ files resulting from next-generation sequencing was performed through the Genome One platform (Dreamgenics, S. L.). The bioinformatics analysis pipeline integrated the following steps: quality check of the FASTQ files using FastQC [68]; removal of bases, adapters, and other low-quality sequences with the fastp tool [69]; alignment against the GRCh38 analysis set version of the reference human genome with bwa-mem [70]; generation of ordered bam files with SAMtools (version 1.22) [71]; removal of optical and PCR duplicates with Sambamba [72]; and identification of SNVs/indels using a combination of VarScan 2 [73] and a proprietary variant-calling algorithm from Dreamgenics. Variants were annotated with information from functional (RefSeq, PFAM), population (dbSNP, 1000 Genomes, ESP6500, ExAC, gnomAD, mtDB), in silico functional impact prediction (dbNSFP, dbscSNV), and clinical (ClinVar, HPO) databases. In terms of the prevalence of the genetic variants studied in our work, we selected a maximum allele frequency (MAF) of 0.05 throughout all analyses, except for the general one found in Section 2.1 (Results), where MAF was 0.01. The MAF threshold was set at 0.05 instead of 0.01 to increase the likelihood of detecting homozygous variants in our sample given the relevance of homozygosity in uncovering recessive variants potentially associated with phenotypic traits. MAF was obtained from 1000 Genomes, ExAC, ESP, gnomADv2 Exomes, gnomADv2 Genomes, and gnomADv3.

All FASTAQ files generated and used for exome analysis have been deposited in NCBI GenBank under BioProject accession number PRJNA1244848.

### 4.7. Gene Ontology (GO) Enrichment Analysis

Gene Ontology (GO) enrichment analysis was performed using the R package *clusterProfiler* (version 4.16.0) within the RStudio environment (R version 4.5.1) on macOS. The analysis was conducted separately for the Biological Process (BP) and Molecular Function (MF) ontologies. The input gene list consisted of 13 human solute carrier (SLC) family genes, derived from Table 2 (homozygous rare variants of the SLC gene superfamily in the patient group) and Table 9 (rare variants in the population shared by several patients and none of the controls in the sample): *SLC4A2*, *SLC6A1*, *SLC6A14*, *SLC9A7*, *SLC13A3*, *SLC13A4*, *SLC35D2*, *SLCO1A2*, *SLC39A10*, *SLC9B1*, *SLC22A25*, *SLC38A6*, and *SPNS2*. Ensembl gene symbols were converted to Entrez Gene IDs using the org.Hs.eg.db annotation package. GO enrichment was performed using the enrichGO() function with the following parameters: *ont* = “*BP*” *or* “*MF*”; *pAdjustMethod* = “*BH*”; *pvalueCutoff* = 0.05; and *qvalueCutoff* = 0.2. The top enriched GO terms were selected based on adjusted *p*-values (Benjamini–Hochberg correction).

### 4.8. Statistical Analyses

Comparisons between groups were conducted using the independent-samples Student’s t-test, allowing for unequal variances unless otherwise stated. Significance levels of *p* < 0.05, marked with *, *p* < 0.01, marked with **, and *p* < 0.001, marked with ***, were considered statistically significant, although the actual *p*-values are indicated whenever possible. All statistical analyses were performed using the SPSS statistic software (version 27.0.1, Macintosh).

## Figures and Tables

**Table 1:** Global variant frequency on whole-exome analysis.

Sample		(a)	(b)	(c)
Patient group	Mean	138,510.83	572.83	1309.67
	S.E.M.	2511.38	13.22	22.48
	n = 6			
Control group	Mean	140,591.40	592.20	1340.20
	S.E.M.	557.57	11.29	15.96
	n = 5			
	t-Student	0.4554	0.2941	0.2968
	*p* value	0.4797 n.s.	0.3053 n.s.	0.3155 n.s.

**Table 2:** Genes with low-frequency variants (<5%) in homozygosis.

Gene Group	Control (n = 5)	Patients (n = 6)	*t*-Test	*p* Value
Group A	0	0		--
Group B	*TRPM2*—SC	0		--
Group C		*SLC4A2*—R		
	*SLC35A2*—NSC	*SLC6A1*—R	−3.9	0.0036 **
		*SLC6A14*—SC		
		SLC9A7—NSC		
		SLC13A3—NSC		
		SLC13A4—NSC		
		SLC35D2—R		
		SLCO1A2—SC		
Group D	0	0		--

** *p* < 0.01; R: change in a regulatory region; SC: synonymous change in the coding region that does not alter the amino acid sequence; NSC: non-synonymous change in the coding region that encodes a different amino acid.

**Table 3:** Low-frequency (<5%) gene variants in heterozygosis for Group A.

Group A Genes	Controls (n = 5)	Patients (n = 6)
*CAT*	0	0
*CCKBR*	1	0
*CYP2D6*	6	1
*FADS1*	0	0
*GSTM1*	0	1
*GSTP1*	0	1
*GSTT1*	0	0
*MTHFR*	0	1
*NAT2*	0	0
*NOS2*	0	0
*NOS3*	2	2
*OPRL1*	0	0
*PON1*	0	0
*PPARG*	0	0
*SCN9A*	1	1
*SIRT1*	2	1
*SOD2*	3	0
Mean ± S.E.M.	3.0 ± 0.71	1.33 ± 0.33
t-Student and *p* value	t = 2.13	*p* = 0.0770 n.s.

**Table 4:** Low-frequency (<5%) gene variants in heterozygosis for Group B.

Group B Genes	Controls (n = 5)	Patients (n = 6)
*TRPA1*	2	1
*TRPC1*	0	0
*TRPC2*	0	0
*TRPC3*	0	1
*TRPC4*	0	2
*TRPC5*	1	0
*TRPC6*	1	0
*TRPC7*	0	0
*TRPM1*	2	4
*TRPM2*	5	2
*TRPM3*	4	4
*TRPM4*	0	2
*TRPM5*	1	4
*TRPM6*	0	0
*TRPM7*	0	1
*TRPM8*	0	0
*MCOLN1*	0	1
*MCOLN2*	1	0
*MCOLN3*	2	0
*PKD1*	6	12
*PKD2*	2	2
*PKD2L1*	2	0
*PKD2L2*	5	4
*TRPV1*	1	1
*TRPV2*	0	0
*TRPV3*	1	3
*TRPV4*	2	2
*TRPV5*	0	0
*TRPV6*	0	2
Mean ± S.E.M.	7.2 ± 1.07	8.0 ± 1.24
t-Student and *p* value	t = −0.49	*p* = 0.6363 n.s.

**Table 5:** Low-frequency (<5%) gene variants in heterozygosis for Group D.

Group D Genes	Controls (n = 5)	Patients (n = 6)
RISK GENES		
*ANKRD6*	2	0
*NALF1 (FAM155A)*	1	1
*WWOX*	2	4
*DAB1*	1	3
*RIMS2*	2	1
*DOCK2*	2	4
*SLC25A38*	1	1
*GIGYF2*	2	2
*BCAS3*	2	2
*STK32B*	2	3
*ECHDC2*	0	0
Mean ± S.E.M.	3.4 ± 0.93	3.5 ± 0.89
t-Student and *p* value	t = −0.08	*p* = 0.9395 n.s.
PROTECTIVE GENES		
*GRIN2A*	0	1
*GNA12*	2	0
*CXCR2P1*	1	2
*LRIG2*	0	1
*TNIK*	1	1
*PAK5 (PAK7)*	0	0
*TSPAN9*	0	0
*ARHGAP22*	3	2
Mean ± S.E.M.	1.4 ± 0.60	1.17 ± 0.48
t-Student and *p* value	t = 0.30	*p* = 0.7686 n.s.

**Table 6:** Low-frequency (<5%) gene variants in heterozygosis for Group C.

Group C Genes	Controls (n = 5)	Patients (n = 6)
291 Gene Variants	192	306
Mean + SEM	46.4 ± 2.84	51.0 ± 1.79
t-Student and *p* value	t = −1.37	*p* = 0.2128 n.s.

**Table 7:** Low-frequency (<5%) gene variants in heterozygosis for Group C (restricted list).

Group C Gene Family	Controls (n = 5)	Patients (n = 6)
*SLC1*	9	10
*SLC4*	7	10
*SLC5*	6	15
*SLC6*	10	10
*SLC7*	5	9
*SLC8*	3	3
*SLC10*	1	4
*SLC16*	1	5
*SLC17*	1	1
*SLC18*	1	0
*SLC22*	7	16
*SLC38*	3	10
Mean ± S.E.M.	10.8 ± 0.20	15.5 ± 0.76
t-Student and *p* value	t = −4.82	*p* = 0.005 **

** *p* < 0.01.

**Table 8:** Variant location for low-frequency gene variants (<5%) in Table 7.

Variant Location	Controls (n = 5)	Patients (n = 6)	t-Student and *p* Value
R	31 (57%)	32 (34%)	0.67 (*p* = 0.53 n.s.)
SC	14 (26%)	34 (37%)	−4.26(*p* = 0.003 **)
NSC	9 (17%)	27 (29%)	−1.88 (*p* = 0.09 n.s.)

** *p* < 0.01; R: change in a regulatory region; SC: synonymous change in the coding region that does not alter the amino acid sequence; NSC: non-synonymous change in the coding region that encodes a different amino acid.

**Table 9:** Rare variants (<5%) in the population shared by several patients in the sample and not by any controls.

Chromosome	Gene	Gene Variant	MAF (in the Population)	Number of Patients
CHR2	*SLC39A10*	rs72913263	0.015916	3
CHR3	*SLC6A1*	rs41293373	0.006309	4 *
CHR4	*SLC9B1*	rs201645894	0.036757	3
		rs369692318	0.029791	4
		rs764250836	0.009533	4
CHR11	*SLC22A25*	rs61930188	0.031116	3
CHR14	*SLC38A6*	rs17097938	0.049403	3
CHR17	*SPNS2 (SLC63A2)*	rs2144402808	0	3

* Appears in 3 patients but in one of them in homozygosis. MAF, maximum allele frequency.

## Data Availability

All sequencing files generated and used for exome analysis have been deposited in the NCBI GenBank under BioProject accession number PRJNA1244848.

## Abbreviations

The following abbreviations are used in this manuscript:

MCS	Multiple chemical sensitivity
SLC	Solute carrier
WES	Whole-exome sequencing
GWAS	Genome-wide association study
SNP	Single-nucleotide polymorphism
